# Community-based distribution of iron–folic acid supplementation in low- and middle-income countries: a review of evidence and programme implications

**DOI:** 10.1017/S1368980017002828

**Published:** 2017-10-24

**Authors:** Justine A Kavle, Megan Landry

**Affiliations:** 1 Maternal and Child Survival Program (MCSP), 455 Massachusetts Ave. NW, Suite 1000, Washington, DC 20036, USA; 2 PATH, Maternal, Newborn, Child Health and Nutrition, Washington, DC, USA; 3 Department of Prevention and Community Health, The George Washington University, Milken Institute School of Public Health, Washington, DC, USA; 4 Independent Consultant, PATH, Washington DC, USA

**Keywords:** Community-based distribution, Iron–folic acid supplementation, Anaemia, Women

## Abstract

**Objective:**

The present literature review aimed to review the evidence for community-based distribution (CBD) of iron–folic acid (IFA) supplementation as a feasible approach to improve anaemia rates in low- and middle-income countries.

**Design:**

The literature review included peer-reviewed studies and grey literature from PubMed, Cochrane Library, LILAC and Scopus databases.

**Setting:**

Low- and middle-income countries.

**Subjects:**

Non-pregnant women, pregnant women, and girls.

**Results:**

CBD programmes had moderate success with midwives and community health workers (CHW) who counselled on health benefits and compliance with IFA supplementation. CHW were more likely to identify and reach a greater number of women earlier in pregnancy, as women tended to present late to antenatal care. CBD channels had greater consistency in terms of adequate supplies of IFA in comparison to clinics and vendors, who faced stock outages. Targeting women of reproductive age through school and community settings showed high compliance and demonstrated reductions in anaemia.

**Conclusions:**

CBD of IFA supplementation can be a valuable platform for improving knowledge about anaemia, addressing compliance and temporary side-effects of IFA supplements, and increasing access and coverage of IFA supplementation. Programmatic efforts focusing on community-based platforms should complement services and information provided at the health facility level. Provision of training and supportive supervision for CHW on how to counsel women on benefits, side-effects, and when, why, and how to take IFA supplements, as part of behaviour change communication, can be strengthened, alongside logistics and supply systems to ensure consistent supplies of IFA tablets at both the facility and community levels.

Globally, anaemia affects 29 % of pregnant women and 38 % of non-pregnant women^(^ ^1^ ^)^ and is associated with one-fifth of maternal deaths^(^ ^2^ ^)^. Anaemia puts women at greater risk of mortality, morbidity, postpartum haemorrhage and poor birth outcomes, including preterm births and low birth weight^(^ ^3^ ^,^ ^4^ ^)^. The WHO recommends daily iron–folic acid (IFA) supplementation (30–60 mg iron, 0·4 g folic acid) initiated as early as possible and continued throughout pregnancy for all adolescent and adult women as a key intervention to reduce the risk of maternal anaemia, iron deficiency and infants born with low birth weight^(^ ^5^ ^)^. According to findings from a recent meta-analysis, IFA supplementation would increase the mean blood Hb concentration by 10·2 (95 % CI 6·1, 14·2) g/l in pregnant women and by 8·6 (95 % CI 3·9, 13·4) g/l in non-pregnant women (aged 19–21 years)^(^ ^6^ ^)^. Applying these shifts to estimated blood Hb concentrations indicates that about 50 % of anaemia in women could be eliminated by IFA supplementation^(^ ^6^ ^)^.

A secondary analysis of national Demographic and Health Survey data sets in nineteen African countries found that when pregnant women received at least ninety IFA supplements through antenatal care (ANC), the risk of neonatal mortality decreased by 34 %^(^ ^7^ ^)^. Similar findings were shown in Nepal, as neonatal mortality decreased by 45 % in the first week and 42 % in the first 28d when women started taking IFA supplements in their first trimester of pregnancy, or if they took at least 150 IFA supplements during their pregnancy^(^ ^8^ ^,^ ^9^ ^)^.

Despite these benefits of maternal IFA supplementation, many low- and middle-income countries continue to face high anaemia rates^(^ ^10^ ^,^ ^11^ ^)^. Interventions delivered at the health facility level, such as IFA supplementation, are not operating at scale in most countries due to lack of demand from health sectors and beneficiaries (e.g. low ANC attendance), limited funding, stock outages and ineffective management of supplies^(^ ^12^ ^–^ ^15^ ^)^.

Although these findings reveal the benefits of IFA supplementation for anaemia and neonatal outcomes, more information is needed on best practices and the most effective strategies to deliver IFA supplementation through community-based channels to complement ANC, since access and supply are issues^(^ ^12^ ^–^ ^15^ ^)^. In the present review, we examine evidence for community-based distribution (CBD) of IFA supplementation as a feasible approach to improve anaemia rates in low- and middle-income countries.

## Design and methods

An extensive literature review of peer-reviewed and grey literature on CBD of IFA supplementation for pregnant women and women of reproductive age (WRA) was conducted. This search strategy was developed and reviewed by the authors and included the following keywords in various combinations: ‘community based distribution’ & ‘IFA’, ‘iron–folic acid’ & ‘community health workers’, ‘CBD of IFA’, ‘iron–folic acid’ & ‘community level’, ‘IFA’ & ‘community utilization’ and/or ‘community’ & ‘iron–folic acid supplements’. We searched published literature, including quantitative, qualitative and mixed-methods studies, from PubMed, Cochrane Library, LILAC and Scopus databases. The initial search returned 147 articles, which were reviewed to determine eligibility for inclusion. Inclusion criteria included studies published between the years of 2000 and 2015 and studies which reported a community element and/or community-based distribution of IFA supplements that described at least one of the following components of programme implementation: type of health worker, supply and demand issues, coverage within the community, and strategies to improve IFA supplementation coverage and utilization for pregnant women and WRA in low- and middle-income countries. CBD of IFA supplementation was reported through various platforms, including private pharmacies within communities, community health centres, home visits from health workers and community gatherings for health education sessions. The type of worker/distributor at the community level varied and included midwives, community health workers (CHW), volunteer health workers, mobile health workers from local health centres, village youth volunteers and pharmacists.

One author reviewed and screened titles and abstracts to determine initial inclusion, while the other author confirmed the final articles for the review. This resulted in a final pool of twenty-two articles with findings from Ending Preventable Maternal and Child Deaths (EPCMD)^*^ priority countries, including Bangladesh, India, Kenya, Mali, Nepal, Pakistan, Senegal and Tanzania, as well as findings from other countries: Cambodia, Iran, Philippines, Tibet and Vietnam (see Table 1). Excluded articles were those without a community component (e.g. IFA supplementation strictly at the health facility level) and reviews of previous programmes (e.g. systematic reviews of programme evaluations).

**Table 1:** Summary of key findings from articles included in the present review of community-based distribution of iron–folic acid supplementation in low- and middle-income countries

Reference	Year	Country	Study description and type of community-based distributor	Key findings: knowledge/awareness, side-effects, IFA counselling, consumption and compliance, anaemia prevalence, ANC attendance
Aguayo *et al.* ^(^ ^25^ ^)^	2005	Mali	IFA supplement distribution to pregnant and lactating women through health workers	∙ Key elements for adherence and effective programming include access and provision of clear information and counselling
Alam *et al*.^(^ ^17^ ^)^	2015	Bangladesh	IFA supplement distribution and counselling through CHW early in pregnancy	∙ Barriers identified included the belief that IFA supplementation would increase fetus size and cause complications, and concerns from decision makers about starting IFA supplementation early in pregnancy
Angeles-Agdeppa *et al*.^(^ ^30^ ^)^	2005	Philippines	Weekly IFA distribution for WRA by physicians, nurses and midwives, assisted by volunteer barangay health workers. Also had a strong social mobilization and marketing component	∙ Awareness of the role of IFA supplementation in anaemia prevention increased to more than 80 % after 12 months ∙ Positive attitudes towards IFA supplementation and knowledge of food sources of iron increased throughout the study ∙ Self-reported compliance increased to over 95 % by the end of 12 months
Bharti^(^ ^34^ ^)^	2004	India	Directly observed home-based twice daily therapy through village youth volunteers	∙ Documented compliance was 87 % ∙ Anaemia prevalence decreased by 40 % in the first 3 months ∙ Occasional side-effects were seen as a barrier by some participants
Bhutta *et al*.^(^ ^35^ ^)^	2009	Pakistan	Randomized fortnightly IFA supplementation or multiple micronutrient distribution to pregnant women through home visits by CHW	∙ Documented compliance in both groups was about 75 % ∙ 10 % fewer LBW infants among women receiving multiple micronutrients compared with IFA supplementation ∙ Side-effects were noted as a barrier
Casey *et al*.^(^ ^40^ ^)^	2009	Vietnam	Weekly distribution of universal IFA supplementation and deworming medication for WRA through village health workers and integration into existing health services	∙ Anaemia prevalence decreased from 14·0 to 5·9 % after 3 months and to 4·5 % after 12 months ∙ Literacy and education levels were associated with compliance
Dickerson *et al*.^(^ ^31^ ^)^	2010	Tibet	Distribution of safe and clean birth kits, newborn hats and blankets, and micronutrient supplements to pregnant women through home and community visits by local health-care workers and laypersons	∙ Nearly 100 % of outreach recipients received micronutrient supplements and safe and clean birth kits
Garcia *et al*.^(^ ^38^ ^)^	2005	Philippines	Marketing and educational programmes from a private pharmaceutical company to promote weekly IFA supplementation for pregnant and non-pregnant women through local health workers and health unit staff	∙ Increased awareness of iron-deficiency anaemia, its causes and effects, and the importance of weekly IFA supplementation
Kanal *et al*.^(^ ^18^ ^)^	2005	Cambodia	Pilot programme of social marketing and community mobilization in secondary-school girls, women working in urban garment factories and women in rural villages. Supplements were distributed or sold by volunteer school-based peer educators, garment factory team leaders and rural peer educators, respectively	∙ Substantial improvements in knowledge about the causes, consequences and prevention of anaemia ∙ Majority of participants showed interest in continuing to take supplements
Khan *et al*.^(^ ^24^ ^)^	2005	Vietnam	Community mobilization and social marketing to promote weekly IFA supplementation in WRA. Supplements were distributed at health stations or were sold through the Women’s Union network	∙ Increased knowledge and participation in preventive weekly IFA supplementation ∙ Purchasing and consuming weekly IFA supplements were between 55 and 92 %
Lutsey *et al*.^(^ ^28^ ^)^	2008	Philippines	Community-based IFA supplementation programme at village health stations through the Philippine iron supplementation programme	∙ Self-reported compliance was 85 %, compliance measured by pill count was 70 % ∙ Self-reported compliance, timing of prenatal care and number of living children were associated with Hb concentration ∙ Forgetfulness in adhering to supplements, side-effects, inconvenience and running out of supplements were seen as barriers
Ndiaye *et al*.^(^ ^32^ ^)^	2009	Senegal	Monthly health promotion sessions for pregnant women through community volunteers	∙ Self-reported intake of iron supplements increased from 43 to 60 % ∙ Community-level distribution of supplements increased from 2·6 to 23·3 % ∙ Increased mean Hb level and significantly reduced the risk of anaemia after 9 months
Nisar *et al*.^(^ ^26^ ^)^	2014	Pakistan	Most IFA supplement users got their supplements from either doctors or paid CHW. CHW provide health education and IFA supplements to pregnant women through home visits	∙ 24 % of women living in urban areas, 19 % of women being visited by a CHW and 12 % of women not being visited by a CHW consumed 90 or more IFA tablets during pregnancy ∙ 25 % of women receiving ANC services consumed 90 or more IFA supplements ∙ On average, IFA supplementation was initiated during the fifth month of pregnancy, with only 5 % of women initiating IFA supplementation during the first trimester
Nisar *et al*.^(^ ^19^ ^)^	2014	Pakistan	Most women get IFA tablets from paid lady health workers, government health facilities, and private clinics or pharmacies	∙ Rural women had less knowledge about the benefits of IFA supplementation than urban women ∙ Forgetting, unavailability of supplements, financial limitations, lack of ANC services, family members’ disapproval, lack of knowledge, side-effects, misconception that IFA supplementation is a form of contraceptive, and discontinuation due to feeling better were seen as barriers
Pal *et al*.^(^ ^20^ ^)^	2013	India	Distribution of IFA supplements to pregnant women through village health workers	∙ Women who received counselling on IFA supplementation from the village health worker had 62 % higher compliance than women who did not receive counselling
Phuc *et al*.^(^ ^36^ ^)^	2009	Vietnam	Weekly distribution of IFA supplements and regular deworming for women aged 15–45 years through village health workers was integrated into the existing health service infrastructure	∙ Full or partial compliance was 85 %
Seck and Jackson^(^ ^42^ ^)^	2009	Senegal	Factors affecting compliance with IFA supplementation in pregnant women	∙ Compliance was 86 % in the treatment group *v*. 48 % in the control group ∙ Side-effects, misconceptions about the length of treatment and forgetting to take IFA were barriers
Shivalli *et al*.^(^ ^37^ ^)^	2015	India	Trials of Improved Practices to improve IFA supplement intake in pregnant women. IFA supplements were either purchased or received through the health-care system	∙ Prevalence of anaemia decreased by 50 % in the intervention group and increased by 2·4 % in the control group ∙ More than 85 % of pregnant women in the intervention group were compliant with IFA tablets, compared with only 38 % of the controls
Srivastava *et al*.^(^ ^22^ ^)^	2015	India	Social mobilization interventions to increase demand for consumption of IFA supplements distributed by health functionaries through a government programme	∙ Motivators for women to take IFA supplements included fear of anaemia risks to mother and child, health benefits, regular follow-up and availability, and inclusion of family members in counselling
Wendt *et al*.^(^ ^27^ ^)^	2015	India	Determinants of IFA supplement receipt from health workers and consumption in pregnant women	∙ IFA supplement availability and attendance at ANC were associated with consumption of IFA tablets for 90 d during pregnancy ∙ Timing of ANC initiation and frequency of ANC attendance were associated with the receipt of IFA tablets ∙ 80·5 % of health facilities had a stock outage of IFA supplements on the day of the survey
Yekta *et al*.^(^ ^23^ ^)^	2008	Iran	Distribution of IFA supplements to pregnant women through health workers at ANC	∙ Women reported low knowledge of IFA supplementation benefits and side-effects ∙ 13 % of women consumed IFA supplements during the first four months of pregnancy, 87 % took IFA supplements through the last five months of pregnancy ∙ Side-effects, family members’ disapproval, and belief that IFA supplementation is not necessary as iron is considered to be sourced only from food were seen as barriers
Young *et al*.^(^ ^29^ ^)^	2009	Tanzania	Comparison of distribution of IFA supplements from health services via nurses *v*. private pharmacies through dispensers (pharmacists)	∙ Dispensers may not always ask about high-risk symptoms, yet have better hours, less waiting time, fewer stock outages, and also carry anthelminthic drugs ∙ Women are likely to visit them earlier in ANC ∙ Male dispensers or health workers may have difficulty counselling mothers ∙ IFA supplementation was generally accepted by women and seen as effective

IFA, iron–folic acid; ANC, antenatal care; CHW, community health workers; WRA, women of reproductive age; LBW, low birth weight.

## Results

### Strengths of community-based distribution of iron–folic acid supplementation

#### Community-based distribution of iron–folic acid supplementation is a valuable platform to increase awareness and knowledge of anaemia and iron–folic acid supplementation

Seven studies reported on increased knowledge and coverage of IFA supplementation through provision of messages and counselling on anaemia and IFA supplementation through community-based channels^(^ ^17^ ^–^ ^23^ ^)^. A study from Iran found that CHW provided counselling on the importance of taking IFA supplements for reducing anaemia. Due to increased awareness and knowledge, pregnant women who received messages from CHW about the benefits of IFA supplementation and potential side-effects adhered to IFA supplements for a significantly longer duration (5–9 months) than women who did not receive messages^(^ ^23^ ^)^. Another study in Cambodia, which reported on the implementation of a weekly IFA supplementation government programme with secondary-school girls (*n* 423), women employed in garment factories (*n* 478) and rural women (*n* 639), showed substantial improvements in knowledge about the causes, consequences and prevention of anaemia following promotion through social marketing strategies^(^ ^18^ ^)^. The programme consisted of public broadcasts, billboards, CHW visiting residents, and programme-related T-shirts and bags distributed to community residents.

A government and private-sector pilot project in Vietnam, which employed community-based social mobilization and social marketing approaches in sites supported by volunteer village health workers, government health facility workers and non-governmental organizations, demonstrated significant increases in the percentage of women with awareness that ‘poor nutrition led to anaemia’, that ‘weekly iron–folic acid supplementation could help to prevent anaemia’, of the need for ‘more iron during pregnancy’ and the role of hookworm infection as a cause of iron-deficiency anaemia (*P*<0·001). The percentage of women who recognized the health effects of anaemia and the health benefits of taking an IFA supplement also increased significantly (*P*<0·001)^(^ ^24^ ^)^. Another study in India registered community-level medical practitioners, increased distribution of IFA tablets, and provided women with correct information and messages about consuming IFA tablets. Programme results indicated an increase in awareness of anaemia at the endline survey to more than 90 % of women, which nearly doubled from the baseline figure (49·2 %). In addition, knowledge that taking IFA supplements during pregnancy can prevent anaemia increased significantly from 12·9 % at baseline to 51·5 % at the endline survey^(^ ^22^ ^)^.

Qualitative data from a study in Pakistan illustrated the value of CBD of IFA supplementation as a platform for communicating the benefits of IFA supplements. One rural mother in Pakistan described her experience: ‘These tablets are good to provide strength to our bodies which are weak during the pregnancy, and also improve the feeling of dizziness; these tablets are good for my health’^(^ ^19^ ^)^. In other country contexts such as Bangladesh, India and Senegal, where women received IFA supplements through community channels such as pharmacists and village health workers, women relayed how taking IFA tablets had improved health benefits such as increasing blood volume, leading to fetal nourishment and compensation for blood loss during delivery^(^ ^17^ ^,^ ^20^ ^,^ ^21^ ^)^. In agreement with these studies, in Mali, mothers who received community-based IFA supplementation messages discussed their experience with taking IFA supplements: ‘I feel healthy’, ‘I feel good’ or ‘I don’t fall sick’, ‘the baby is/stays healthy’ and ‘the baby breast-feeds well/a lot/frequently’^(^ ^25^ ^)^.

#### Community-based distribution of iron–folic acid supplementation can encourage attendance to antenatal care

CBD of IFA supplementation can also be an important mechanism to complement ANC, to encourage early and frequent attendance at ANC, and to achieve the WHO recommendation of at least four visits during pregnancy. Late presentation to ANC, in the second or third trimester, and utilization of health services is a key challenge to maternal IFA supplementation provided through ANC^(^ ^23^ ^,^ ^24^ ^,^ ^26^ ^,^ ^27^ ^)^. For example, in the Philippines, the first prenatal visit occurred at 3·80 (sd 1·56) months and mothers averaged less than one visit per month after the initial visit^(^ ^28^ ^)^. Similarly, in another study conducted in Pakistan, maternal IFA supplementation was initiated, on average, in the fifth month of pregnancy, and only 5 % of women presented to ANC and received IFA supplements during their first trimester of pregnancy^(^ ^26^ ^)^. Moreover, one-third of participants in Pakistan did not use ANC services at all during their last pregnancy.

In Pakistan, the Philippines, Nepal, Tanzania and Thailand, distribution of IFA supplementation through community-based channels, such as CHW and various women’s social networks, was found to reach a greater proportion of women compared with ANC^(^ ^17^ ^,^ ^23^ ^,^ ^26^ ^,^ ^29^ ^)^. Six studies found that CBD of IFA supplementation can increase ANC attendance through community agents encouraging earlier and consistent ANC visits^(^ ^25^ ^,^ ^26^ ^,^ ^28^ ^,^ ^30^ ^–^ ^32^ ^)^. In Nepal, a programme with community volunteers that distributed IFA supplements found a substantial increase in compliance (defined as those taking 80 % of the recommended number of supplements) and increased ANC attendance through community volunteers, which dispelled a common local belief that community distribution would discourage women from seeking care at health facilities^(^ ^33^ ^)^.

#### Community-based distribution of iron–folic acid supplementation can increase compliance and address side-effects

Fourteen^(^ ^17^ ^,^ ^18^ ^,^ ^20^ ^,^ ^21^ ^,^ ^24^ ^,^ ^25^ ^,^ ^27^ ^,^ ^28^ ^,^ ^31^ ^,^ ^34^ ^–^ ^37^ ^,^ ^39^ ^)^ of twenty-six studies identified CBD platforms as being successful in addressing factors related to compliance, such as maintaining the daily regimen of one pill per day, temporary side-effects (e.g. vomiting, nausea, dizziness) and forgetfulness. In addition, eight studies reported that more than 75 % of women had high compliance (taking ≥70 % of tablets) with IFA supplementation when there was a consistent supply of IFA supplements from the community level, either with or without IFA supplements delivered through health facilities^(^ ^21^ ^,^ ^24^ ^,^ ^25^ ^,^ ^28^ ^,^ ^34^ ^–^ ^37^ ^)^.

In India, compliance was higher (62 %) among mothers who were counselled by health workers on when, how and why IFA supplementation is important than among those who did not receive guidance^(^ ^20^ ^)^. In Vietnam, a free monthly distribution of IFA supplements indicated that 85 % of WRA achieved full or partial compliance (defined as taking some but not all tablets) to weekly IFA supplementation through the existing health service infrastructure (e.g. health clinics and facilities) with village health workers as the direct point of contact; and included training for village health workers on anaemia, IFA supplementation and deworming^(^ ^36^ ^)^. In a randomized study in Senegal, midwives were a strong motivator for improved IFA supplementation compliance in the treatment group (86 %) *v.* the control group (48 %; *P*<0·0001), as midwives encouraged women to take IFA tablets by influencing their perceptions that IFA tablets would improve health and reduce anaemia^(^ ^21^ ^)^.

In addition to the findings above, seven studies described the use of social marketing, counselling and health education methods, in combination with CBD, to increase access and compliance to IFA supplementation^(^ ^17^ ^,^ ^18^ ^,^ ^24^ ^,^ ^25^ ^,^ ^27^ ^,^ ^31^ ^,^ ^38^ ^)^. In Vietnam, rates of buying and consuming a weekly IFA supplement for WRA in programme sites were 55 and 92 %, respectively. High rates were attributed to increased knowledge from community-based social marketing and mobilization^(^ ^24^ ^)^. In another study carried out in Pakistan, lady health workers, who conduct routine home visits, positively influenced increased consumption of IFA supplements, as 19 % of women residing in programme areas consumed ninety or more tablets, compared with only 12 % in non-programme areas^(^ ^26^ ^)^.

Community workers aided women to comply with IFA supplementation throughout pregnancy through home visit reminders, as forgetfulness to take the supplements on a daily basis was reported as a primary reason for non-compliance in settings such as India, Mali, Pakistan, the Philippines and Senegal^(^ ^19^ ^,^ ^21^ ^,^ ^25^ ^,^ ^28^ ^)^. Five studies circumvented forgetfulness by utilizing village health volunteers to encourage mothers to use ANC and visiting homes to provide reminders for taking pills^(^ ^20^ ^,^ ^31^ ^,^ ^34^ ^–^ ^36^ ^)^. Moreover, in India, Tibet and Nicaragua, community health volunteers and other community-level workers delivered supplements and provided clients with follow-up counselling, which helped women understand how to address potential and temporary side-effects such as vomiting, nausea and dizziness^(^ ^22^ ^,^ ^31^ ^,^ ^39^ ^)^. These strategies often resulted in significantly higher IFA supplement consumption among mothers who received an explanation on IFA supplements from CHW compared with those who were not provided information by the health worker (*χ* ^2^=4·529; *P*<0·05)^(^ ^20^ ^)^.

### Barriers to successful roll-out of community-based distribution of iron–folic acid supplementation

#### Advice from influential family and community members

Four articles identified advice from influential family members as a barrier to consumption of the IFA supplements^(^ ^17^ ^,^ ^19^ ^,^ ^22^ ^,^ ^23^ ^)^. One woman reported her mother-in-law’s response when she perceived the iron tablets were causing her to feel ill: ‘I used these [IFA] tablets but after few days I had vomiting and diarrhoea with these [supplements] and my mother-in-law told me to stop this medicine; she [mother-in-law] told me not to take any medicine during pregnancy’^(^ ^19^ ^)^. Similarly, in Iran, although most women adhered to IFA for a 5–9-month period, 13 % of women surveyed stopped taking IFA supplements early, because relatives advised them to stop^(^ ^23^ ^)^. CBD can be used to help alleviate potentially negative advice from family members. Using an example from Tibet as to how programmatically this can be achieved, the Pregnancy and Village Outreach Tibet (PAVOT) programme conducted comprehensive community and home-based maternal newborn and nutrition outreach to rural pregnant women and family members on anaemia and IFA supplementation, as well as antenatal/postpartum care, birth planning, danger sign recognition, clean and safe delivery practices, and breast-feeding^(^ ^31^ ^)^. The PAVOT programme included training of master trainers, who then trained outreach providers comprising laypersons and health-care workers, through role playing, hands-on skills, and distribution of IFA supplements and counselling on their use. Skills included identification of barriers and solutions to reinforce key messages to women and their families. The programme reported that 68 % of programme participants, consisting of pregnant women and family members, received three or more home visits by CHW that entailed counselling and support to address seeking ANC early, antenatal nutrition, micronutrient supplementation and safe delivery practices^(^ ^31^ ^)^. Through the programme, 99 % of pregnant women received IFA supplements, but the programme did not assess compliance to IFA supplementation^(^ ^31^ ^)^.

#### Supplies of iron–folic acid supplements: availability at health facilities *v*. community

Unavailability of IFA tablets at local health facilities was cited as a barrier to compliance in four articles^(^ ^22^ ^,^ ^25^ ^–^ ^27^ ^)^, and seven articles reported high compliance (above 75 %) to IFA supplementation when there was a consistent supply of IFA supplements available to them^(^ ^24^ ^,^ ^25^ ^,^ ^28^ ^,^ ^34^ ^–^ ^37^ ^)^. For example, in India, adequate IFA supplement supply was significantly associated with increased IFA supplement consumption when controlling for demographic variables (OR=1·33; 95 % CI 1·03, 1·71)^(^ ^27^ ^)^. Women residing in villages where a health centre had available supplies of IFA supplements were more likely to have consumed IFA tablets for ninety or more days during their last pregnancy (OR=1·37; 95 % CI 1·04, 1·82)^(^ ^27^ ^)^.

Findings from a few studies revealed that stock outages at the health facility level were more frequently reported as a barrier than side-effects (e.g. constipation and nausea)^(^ ^22^ ^,^ ^27^ ^)^. Community channels, such as private pharmacies, midwives and community agents, were more likely to have consistent supplies of IFA supplements compared with clinics and hospitals, who faced stock outages^(^ ^29^ ^,^ ^38^ ^)^. In one study, it was noted that women will ‘only sometimes’ purchase IFA supplements from a pharmacy with a prescription when community-based lady health workers and/or health facilities faced stock outages of IFA supplements^(^ ^26^ ^)^.

#### Cost in relation to compliance

Six studies reported IFA supplementation was provided free of charge through CBD^(^ ^17^ ^,^ ^19^ ^,^ ^25^ ^,^ ^32^ ^,^ ^36^ ^,^ ^38^ ^)^. A few studies assessed the impact of cost in relation to compliance and in relation to purchasing IFA tablets through private pharmacies. In Senegal, a study found significantly higher compliance (86 %) when midwives distributed free IFA tablets to pregnant women after their initial ANC visit at a health facility, compared with women receiving a prescription to purchase the tablets from a private pharmacy or community vendor for $US 0·01 for ten tablets (48 %), indicating that when women are expected to purchase the tablets, compliance may be lessened^(^ ^21^ ^)^. In Cambodia, supplements were sold to women for $US 0·01 for one month’s supply (four tablets) and peer educators went door-to-door to educate and promote the supplements in rural villages, whereas in two other study settings (factories and schools), IFA tablets were provided free of charge. Compliance, defined as adhering to a weekly regimen, as reported by women in each of the three settings, was 55 % for schoolgirls, 57 % for female factory workers and 71 % for rural WRA^(^ ^18^ ^)^, indicating the sale of tablets, along with the peer education, proved to be the most effective in getting women to consume IFA supplements.

### Impact of community-based distribution of iron–folic acid supplementation: coverage and reductions in maternal anaemia

Targeting pregnant women and WRA through community settings demonstrated increased accessibility, high compliance, and reductions in anaemia in thirteen studies^(^ ^20^ ^,^ ^23^ ^,^ ^24^ ^,^ ^25^ ^,^ ^28^ ^,^ ^30^ ^,^ ^34^ ^–^ ^37^ ^,^ ^40^ ^,^ ^42^ ^)^. Nicaragua increased IFA supplementation coverage among pregnant women to over 80 % and experienced a substantial drop in anaemia prevalence through use of community-based distributors who provided counselling and follow-up to pregnant women^(^ ^39^ ^)^. A study that applied the Trial of Improved Practices (TIPs) methodology in India aimed to increase positive perceptions of IFA supplementation, IFA supplementation uptake and dietary practices^(^ ^37^ ^)^. Results of that study indicated that the prevalence of anaemia was reduced by half in the TIPs group and increased by 2·4 % in the control group^(^ ^37^ ^)^. In Senegal, CBD of iron supplements, alongside implementation of monthly healthy pregnancy promotion sessions delivered via community volunteers, improved accessibility and significantly reduced anaemia prevalence from 85 to 55 % between baseline and endline (*P*<0·0001) in the positive deviant intervention group, which was significantly different from the control group not receiving the positive deviant approach (*P*=0·003)^(^ ^32^ ^)^.

In another study, a free weekly IFA supplementation programme in Vietnam assessed effects on anaemia levels. Weekly IFA supplementation and four monthly deworming tablets were distributed through the existing health structure, where all WRA were encouraged to collect packs of four ferrous sulfate/folic acid tablets (60 mg/0·4 mg) from their village health worker each month^(^ ^40^ ^)^. At 3 months post-implementation, anaemia reduced to 5·9 % (relative risk=0·43; 95 % CI 0·26, 0·70; *P*=0·001); and after 12 months, anaemia levels were further reduced to 4·5 % (relative risk=0·32; 95 % CI 0·15, 0·68; *P*=0·003)^(^ ^40^ ^)^. Similarly, a community-based programme in India reported a significant overall decrease in anaemia between baseline and endline from 72·6 to 50·7 % (*P*<0·001) through the use of registered medical practitioners at the community level to provide women with information, tablets and messaging around consuming IFA tablets^(^ ^22^ ^)^.

## Discussion

To our knowledge, the present review is the first which has assessed the effectiveness, strengths and challenges of CBD of IFA supplementation via a programmatic perspective relevant to low- and middle-income countries. The strength of the review lies in the compilation of data on CBD of IFA supplementation as a valuable and potential platform for reducing anaemia and increasing ANC coverage and access, which included increases in awareness and knowledge, compliance and coverage of IFA supplementation for pregnant women and WRA. CBD of IFA supplementation showed success in reducing anaemia with community-based health workers or volunteers who counselled on health benefits, side-effects and compliance with IFA supplementation. These findings are consistent with other research that found community-level workers or volunteers to be instrumental in educating women about common side-effects and how to manage side-effects in order to increase compliance^(^ ^27^ ^,^ ^41^ ^)^.

The present review also highlights that CBD of IFA supplementation is a potential platform for encouraging earlier and frequent attendance at ANC, as community-level workers were more likely to identify and reach a greater number of women earlier in pregnancy because women tended not to present to ANC until after the first trimester^(^ ^27^ ^,^ ^28^ ^,^ ^33^ ^)^. Thus, targeted community distribution could be a successful strategy to not only encourage women to go for earlier ANC visits, but also to start women on an IFA supplementation regimen earlier in their pregnancy^(^ ^29^ ^)^.

Several potential challenges to CBD of IFA supplementation exist. Women reported IFA tablets were more frequently available from CBD channels, such as community vendors or community workers, as compared with health facilities that face stock outages^(^ ^29^ ^,^ ^38^ ^)^. However, inventory systems would be required to forecast and monitor IFA supplies at the community level. Logistics, storage and distribution of IFA supplements (ninety or more supplements per pregnant woman) could be bulky and cumbersome for community workers to provide during household visits.

Several studies have provided strong recommendations for IFA supplementation to be free of charge at the community and facility levels for increased utilization and compliance^(^ ^24^ ^,^ ^36^ ^,^ ^42^ ^,^ ^43^ ^)^. Our findings indicate that the availability and accessibility of free or low-cost commodities improved the use of antenatal IFA supplements. However, even when free of charge, distribution was still cited as a barrier due to frequent stock outages, and this was consistent with other reviews^(^ ^19^ ^,^ ^44^ ^)^. It was also noted that women who live far from government health clinics or outside the CHW service area have a difficult time obtaining free IFA tablets and often cannot afford to purchase them from a private pharmacy^(^ ^19^ ^)^. Private distribution points and pharmacies often have associated costs that may limit accessibility and/or desire for IFA supplements^(^ ^30^ ^)^. However, some women considered paying for and the price of IFA tablets to be acceptable^(^ ^24^ ^)^, and others would be willing to purchase the tablets after free distribution programmes ended^(^ ^18^ ^)^.

Our findings revealed that counselling on IFA supplementation could be strengthened through community-based distributors who provide consistent and clear messages on IFA supplementation, as raising awareness and increasing knowledge of IFA and anaemia are critical. Key factors for successful CBD of IFA supplementation programmes include ensuring adequate supply of the IFA commodities, strengthening mechanisms for CBD to increase access for women, provision of training and supervision for CHW on why, how and when IFA should be given, in addition to preparing mothers on how to manage any potential, yet temporary, side-effects (i.e. constipation, black stool), and promotion of behaviour change communications through culturally relevant key messages and counselling in order to increase demand for and compliance with IFA supplementation^(^ ^45^ ^)^. Engagement with professional associations, such as local nursing, midwifery and physician associations, may be valuable as stakeholders to promote inclusion of CBD of IFA supplementation in national policies and programmes.

### Limitations

The current review has several limitations. Information on the role of governance (i.e. public sector-supported CHW, dedicated policies on CBD of IFA supplementation) in relation to community-based platforms was not collected or provided in the studies included in the review. Information on CBD of IFA supplementation consists only of information provided in the current reviewed studies, which often lacked specific data on the IFA supplementation counselling that was received and seldom reported on the specific messages. Only fourteen studies reported data on compliance with IFA supplementation regimens, and few studies reported programme coverage and impact on anaemia.

## Conclusions

CBD of IFA supplementation can be a valuable platform for increasing awareness, improving knowledge, addressing compliance and side-effects, and increasing access and coverage of IFA supplementation. Programmatic efforts should focus on community-based platforms that complement services at the health facility level. Provision of training and supportive supervision for community-level agents on how to counsel women on benefits and side-effects and when, why and how to take IFA supplements, as part of behaviour change communication, should be strengthened, alongside logistics and supply systems to ensure consistent supplies of IFA tablets.

## Footnotes

* The US Agency for International Development’s (USAID’s) 2014 *Acting on the Call* Report formulated country-specific plans for working with partners in twenty-four priority countries to save the lives of mothers and children. The follow-up 2015 *Acting on the Call* Report provided country-by-country progress updates with new recommendations for reaching 38 million women with increased access to high-quality health services around the time of delivery. USAID’s 2016 *Acting on the Call* Report built on the 2014 and 2015 Reports with new updates and preliminary data on progress made over the past year in twenty-four priority countries and introduced Burma as the twenty-fifth priority country^(^ ^16^ ^)^.
